# IL-13 signaling via IL-13Rα_2_ triggers TGF-β_1_-dependent allograft fibrosis

**DOI:** 10.1186/2047-1440-2-16

**Published:** 2013-10-22

**Authors:** Stefan M Brunner, Gabriela Schiechl, Rebecca Kesselring, Maria Martin, Saidou Balam, Hans J Schlitt, Edward K Geissler, Stefan Fichtner-Feigl

**Affiliations:** 1 Department of Surgery, University Medical Center Regensburg, Franz-Josef-Strauss-Allee 11, Regensburg 93053, Germany; 2 Regensburg Center of Interventional Immunology, University Medical Center Regensburg Regensburg, Germany

**Keywords:** IL-13, IL-13Rα_2_, TGF-β_1_, Allograft fibrosis, Heart transplantation

## Abstract

**Background:**

Allograft fibrosis still remains a critical problem in transplantation, including heart transplantation. The IL-13/TGF-β_1_ interaction has previously been identified as a key pathway orchestrating fibrosis in different inflammatory immune disorders. Here we investigate if this pathway is also responsible for allograft fibrosis and if interference with the IL-13/TGF-β_1_ interaction prevents allograft fibrosis.

**Methods:**

FVB or control DBA/1 donor hearts were transplanted heterotopically into DBA/1 recipient mice and hearts were explanted at day 60 and 100 post-transplantation. Cardiac tissue was examined by Masson’s trichrome staining and immunohistochemistry for CD4, CD8, CD11b, IL-13, Fas ligand, matrix metalloproteinase (MMP)-1, MMP-13, β2-microglobulin, and Gremlin-1. Graft-infiltrating cells were isolated and analyzed by flow cytometry. IL-13 and TGF-β_1_ levels were determined by enzyme-linked immunosorbent assay (ELISA) and the amount of collagen was quantified using a Sircol assay; IL-13Rα_2_ expression was detected by Western blotting. In some experiments IL-13/ TGF-β_1_ signaling was blocked with specific IL-13Rα_2_ siRNA. Additionally, a PCR array of RNA isolated from the allografts was performed to analyze expression of multiple genes involved in fibrosis.

**Results:**

Both groups survived long-term (>100 days). The allogeneic grafts were infiltrated by significantly increased numbers of CD4^+^ (*P* <0.0001), CD8^+^ (*P* <0.0001), and CD11b^+^ cells (*P* = 0.0065) by day 100. Furthermore, elevated IL-13 levels (*P* = 0.0003) and numbers of infiltrating IL-13^+^ cells (*P* = 0.0037), together with an expression of IL-13Rα_2_, were detected only within allografts. The expression of IL-13 and IL-13Rα_2_ resulted in significantly increased TGF-β_1_ levels (*P* <0.0001), higher numbers of CD11b^high^Gr1^intermediate^TGF-β_1_^+^ cells, and elevated cardiac collagen deposition (*P* = 0.0094). The allograft fibrosis found in these experiments was accompanied by upregulation of multiple profibrotic genes, which was confirmed by immunohistochemical stainings of allograft tissue. Blockage of the IL-13/TGF-β_1_ interaction by IL-13Rα_2_ siRNA led to lower numbers of CD11b^high^Gr1^intermediate^TGF-β_1_^+^, CD4^+^, CD8^+^, and CD11b^+^ cells, and prevented collagen deposition (*P* = 0.0018) within these allografts.

**Conclusions:**

IL-13 signaling via IL-13Rα_2_ induces TGF-β_1_ and causes allograft fibrosis in a murine model of chronic transplant rejection. Blockage of this IL-13/TGF-β_1_ interaction by IL-13Rα_2_ siRNA prevents cardiac allograft fibrosis. Thus, IL-13Rα_2_ may be exploitable as a future target to reduce allograft fibrosis in organ transplantation.

## Background

Heart transplantation is an effective therapy for chronic heart failure [1]. Recent immunosuppressive strategies have reduced acute rejection episodes and improved early cardiac graft survival [2]. However, these improvements did not ameliorate chronic allograft rejection, which remains an obstacle for better long-term heart transplant survival [3]. Chronic rejection of an allograft causes an intimal fibrosis in the vessels that leads to cardiac allograft vasculopathy [4]. Another consequence of chronic rejection and inflammation is cardiac fibrosis accompanied by increased stiffness of the heart and diminished contractility [5]. Ultimately, these fibrotic reactions can result in myocardial infarction or sudden death [4,6].

On a molecular level, fibrosis is associated with a disruption of the extracellular matrix and with deposition of extracellular collagen produced by myofibroblasts [5]. In various studies TGF-β_1_ has been identified as the key cytokine orchestrating fibrosis development [7,8]. TGF-β_1_ is produced by macrophages after stimulation by IL-13 via the IL-13Rα_2_ in the presence of IL-4 or TNF-α [9]. Further studies have shown that this pathway is a key initiation point for a complex fibrotic program in chronic TNBS colitis [10]. Additionally, it has been demonstrated that TGF-β_1_ inhibition ameliorates lung fibrosis, chronic allograft nephropathy, and also cardiac allograft fibrosis [11-13]. However, no effective therapy to prevent heart allograft fibrosis has been identified so far, possibly because ideal murine transplant models have been lacking to study potential targets and therapies. A mouse model for the examination of cardiac allograft fibrosis should enable long-term survival of a transplanted allograft and develop cardiac fibrosis in the setting of chronic rejection. Tanaka *et al.* have developed a transplantation model in which FVB (H-2q) donor hearts were placed into DBA/1 recipients that display a similar major histocompatibility complex (MHC) (H-2q), but different non-MHC genes (CD5, CD8a, NK1.1, and Thy-1) [14]. In this model, the heart allografts survive for up to more than 100 days without immunosuppression and developed graft coronary artery disease as the result of chronic rejection.

The present study was performed under the hypothesis that the FVB to DBA/1 model is appropriate to examine cardiac graft fibrosis. Further, we hypothesized that TGF-β_1_ stimulated by IL-13 signaling through IL-13Rα_2_ is responsible for this allograft fibrosis and that blockage of the pathway by IL-13Rα_2_-specific siRNA can ameliorate allograft fibrosis.

## Materials and methods

### Mice and heterotopic heart transplantation

Female DBA/1 (H-2q), FVB (H-2q), and as controls BALB/c and C57BL/6 mice, 10 to 12 weeks old, were purchased from The Jackson Laboratory (Bar Harbor, ME, USA) and housed at our local animal care facility. Animal use adhered to institutional guidelines.

Vascularized cardiac allografts were transplanted into the abdomen using a microsurgical technique as previously described by Corry *et al.*[15]. Donor hearts were perfused via the abdominal vena cava and additionally via the aortic arch with cold 0.9% saline (3 mL each) containing 500 IE heparin. Graft function was assessed by palpation of the abdomen and rejection was defined as cessation of cardiac contractility. All donor hearts had palpable contractions at the time of recovery (60 or 100 days; acute rejection 8 days).

### IL-13Rα_2_-specific siRNA

IL-13Rα_2_-specific siRNA and control (scrambled) siRNA for use in gene silencing studies were obtained from Dharmacon (Chicago, IL, USA). The siRNA (100 μg) was encapsulated in HVJ-E and prepared as previously described before administration by intraperitoneal injection (100 μL) every other day [10,16]. The sequence used for the siRNA is 5′-GGAATCTAATTTACAAGGA-3′.

### Histology and immunohistochemistry

Formalin-fixed and paraffin-embedded samples were prepared and sectioned (2 to 3 μm). Tissue sections were stained with Masson’s trichrome. Frozen sections (2 to 3 μm) were blocked with 1% BSA (Biomol, Hamburg, Germany), 10% goat serum (Sigma-Aldrich, St Louis, MO, USA), or an antibody dilution buffer. As primary antibodies, rat monoclonal anti-mouse CD11b (557395; BD, Heidelberg, Germany), CD4 (550280; BD), and CD8 antibodies (Ab25478; Abcam, Cambridge, UK), a goat polyclonal anti-mouse IL-13 antibody (AF-413-NA; R&D Systems, Minneapolis, MN, USA) and a rabbit polyclonal anti-mouse Fas ligand (Ab15285; Abcam), a rabbit polyclonal anti-mouse matrix metalloproteinase (MMP)-1 (orb101432; Biorbyt, Cambridge, UK), a rabbit polyclonal anti-mouse MMP-13 (Ab39012; Abcam), a rabbit polyclonal anti-mouse β2-microglobulin (Ab87483; Abcam), and a rabbit polyclonal anti-mouse Gremlin-1 antibody (Ab90670; Abcam) were used. After staining with goat anti-rat-Fab2 (sc-3822; Santa Cruz Biotechnology, Heidelberg, Germany), donkey anti-goat-Fab2 (sc-2042; Santa Cruz), or goat anti-rabbit-Fab2 (Ab64256; Abcam) secondary antibody, sections were incubated with SensiTek HRP (ScyTec Laboratories, Logan, UT, USA) and positive signals were visualized using a 3,3′-diaminobenzidine-tetrahydrochlorhydrate (DAB) kit (Merck, Darmstadt, Germany) or AEC+ High Sensitivity Substrate Chromogen kit (Dako, Hamburg, Germany). Images were captured using an Axio Observer Z1 microscope (Carl Zeiss, Oberkochen, Germany). For quantifying graft-infiltrating leukocytes, three high power fields (HPFs; 20x magnification) were counted per slide by two independent examiners.

### Western blot analyses

Cells were lysed with radioimmunoprecipitation assay buffer and the whole cell lysates obtained were subjected to SDS-PAGE. The separated proteins obtained were transferred to a nitrocellulose membrane and immunoblotted. IL-13Rα_2_ was detected by incubation with a monoclonal rat anti-mouse IL-13Rα_2_ (R&D Systems), followed by incubation with horseradish peroxidase-conjugated anti-rat IgG (Invitrogen, Carlsbad, CA, USA). Membranes were developed with SuperSignal West Pico Chemiluminescent Substrate (Pierce Chemical, Dallas, TX, USA) and exposed to X-ray film.

### Collagen assay

Heart allografts were harvested on day 60 and day 100 after transplantation, and homogenized in 0.5 mol/L acetic acid containing pepsin (at a concentration of 10 mg tissue/10 mL of acetic acid solution). The resulting mixture was then incubated and stirred for 24 hours at 4°C. Total soluble collagen content of the mixture was then determined with a Sircol Collagen Assay kit (Biocolor, Carrickfergus, UK), as described by the manufacturer. Acid soluble type I collagen supplied with the kit was used to generate a standard curve.

### Cell isolation from cardiac grafts and spleens

Cardiac tissue was minced in 10 mL of RPMI 1640 medium with 10% FCS, 600 U/mL collagenase II (Roche Diagnostics, Mannheim, Germany), and deoxyribonuclease I (DNase; Sigma-Aldrich). This mixture was shaken at room temperature for 2 hours and supernatant was flushed through a 100 μm nylon cell strainer (Schubert & Weiss, Munich, Germany). Remaining tissue was again digested in 5 mL of RPMI-collagenase-DNase solution at 37°C and strained through a 100 μm nylon strainer. Splenic tissue was minced and strained through a 100 μm nylon strainer. Digested cell suspensions were centrifuged for 5 minutes at 1,500 rpm (4°C). To remove red blood cells, the pellet was treated with ACK lysis buffer (Lonza Walkersville, Walkersville, MD, USA) and incubated for 2 minutes at room temperature. After centrifugation, cells were suspended in HBSS medium (Gibco, Grand Island, NY, USA) and counted.

### Flow cytometry

Cell isolates were blocked with 1% mouse serum (Dako, Glostrup, Denmark) and stained with appropriate non-overlapping conjugated monoclonal antibodies (anti-Gr1 antibody from Miltenyi Biotec, Bergisch Gladbach, Germany; all other antibodies from eBioscience, San Diego, CA, USA). Intracellular staining was carried out by first fixing and permeabilizing cells with Cytofix/Cytoperm solution (BD Pharmingen, San Diego, CA, USA). Analyses were performed using a FACSCanto II flow cytometer (BD Biosciences, San Jose, CA, USA). Data were obtained using BD CellQuest Pro acquisition software (BD Biosciences) and analyzed via FlowJo software (Tree Star Inc, Ashland, OR, USA).

### ELISA

Heart allografts were harvested at day 60 and day 100, and graft-infiltrating cells were isolated. Isolated graft-infiltrating cells were cultured at 37°C. For IL-13, we cultured 1 × 10^6^ cells per 1 mL medium for 48 hours; for TGF-β_1_ measurements, we cultured 1 × 10^5^ cells per 100 μL medium for 24 hours. During the culture period cells were stimulated with plate-bound anti-CD3 antibody (10 μg/mL) and soluble anti-CD28 antibody (1 μg/mL; BD Biosciences Pharmingen) for measurement of IL-13 (R&D Systems). For determination of TGF-β_1_ levels (Invitrogen) cells were stimulated with plate-bound anti-CD3 antibody (10 μg/mL), soluble anti-CD28 antibody (1 μg/mL), and recombinant murine IL-13 (20 ng/mL; R&D Systems). Cytokine concentrations were determined in duplicate by enzyme-linked immunosorbent assay (ELISA) according to the manufacturer’s instructions. TGF-β_1_ was measured in medium containing TGF-β_1_-depleted human serum.

### RNA isolation and PCR array

In heart allografts recovered on day 100 after transplantation, RNA was extracted using the RNeasy Mini Kit (Qiagen, Hilden, Germany), as described by the manufacturer. One microgram of total RNA was reverse transcribed using the AffinityScript QPCR cDNA Synthesis Kit (Agilent Technologies, Böblingen, Germany). Expression of genes relevant for fibrosis was determined with a Mouse Fibrosis RT^2^ Profiler PCR Array (SA Biosciences, Hilden, Germany) using the LightCycler 480 Real-Time PCR System (Roche).

### Statistics

All data, unless otherwise specified, are shown as the mean ± standard error of the mean (SEM), and were compared using a two-tailed Student’s test. The level of significance was set at a probability of *P* <0.05.

## Results

### FVB allografts transplanted in DBA/1 recipients showed significantly increased infiltration by CD4^+^, CD8^+^, and CD11b^+^ cells

**Figure 1:**
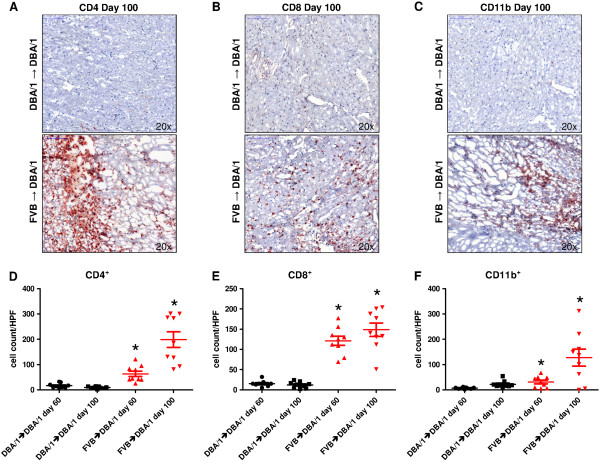
**Increased infiltration by CD4**^**+**^**, CD8**^**+**^**, and CD11b**^**+ **^**cells in allogeneically transplanted grafts. (A,B,C)** Representative stainings for CD4, CD8, and CD11b in syngeneic (DBA/1 into DBA/1) or allogeneic (FVB into DBA/1) heart allografts explanted at day 100 after transplantation. **(D)** In FVB into DBA/1 transplanted hearts, significantly elevated numbers of CD4^+^ cells were detected (day 60, *P* = 0.0007 and day 100, *P* <0.0001; day 60 versus day 100, *P* = 0.0009) in comparison to DBA/1 into DBA/1 transplanted grafts. **(E)** FVB allografts transplanted into DBA/1 recipients showed numbers of CD8^+^ cells (day 60, *P* <0.0001 and day 100, *P* <0.0001; day 60 versus day 100, *P* = 0.1921) that were significantly higher than in the syngeneic group. **(F)** Significantly higher levels of CD11b^+^ cells were found in allogeneic (FVB into DBA/1) grafts (day 60, *P* = 0.0045 and day 100, *P* = 0.0065; day 60 versus day 100, *P* = 0.0124) when compared to DBA/1 to DBA/1 transplanted hearts. The histological score is the mean of 3 HPF (20× magnification); at least five mice per group were analyzed. *P <0.05. HPF, high power field.

To determine the number of graft-infiltrating cells, heart allografts were harvested on day 60 and day 100 after transplantation, and were stained for CD4, CD8, and CD11b. In syngeneic grafts (DBA/1 into DBA/1), low numbers of CD4^+^ (day 60, 16 ± 3 and day 100, 9 ± 1 cells/HPF), CD8^+^ (day 60, 15 ± 2 and day 100, 12 ± 3 cells/HPF), and CD11b^+^ cells (day 60, 6 ± 1 and day 100, 22 ± 5 cells/HPF) were detected (Figure 1A,B,C,D,E,F). Allogeneic heart grafts (FVB into DBA/1) at day 60 after transplantation showed significantly higher cell numbers of CD4^+^ (63 ± 11 cells/HPF; *P* = 0.0007), CD8^+^ (121 ± 11 cells/HPF; *P* <0.0001), and CD11b^+^ cells (31 ± 7 cells/HPF; *P* = 0.0045) compared to grafts in the syngeneic group. The numbers of CD4^+^ and CD11b^+^ cells in the FVB into DBA/1 group increased further by day 100 after transplantation (day 60 versus day 100, CD4^+^*P* = 0.0009; CD11b^+^*P* = 0.0124), whereas the increase in the number of CD8^+^ cells did not reach statistical significance (*P* = 0.1921). In comparison to control animals at day 100 after transplantation, the allogeneic group showed significantly higher levels of CD4^+^ (199 ± 31 cells/HPF; *P* < 0.0001), CD8^+^ (149 ± 17 cells/HPF; *P* <0.0001), and CD11b^+^ cells (128 ± 34 cells/HPF; *P* = 0.0065).

### FVB allografts transplanted into DBA/1 recipients showed significantly increased levels of IL-13, IL-13Rα_2_, and TGF-β_1_

**Figure 2:**
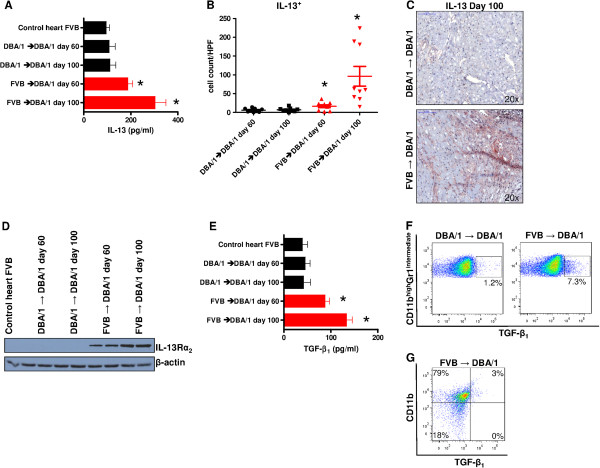
**Activation of IL-13/TGF-β**_**1 **_**pathway in allogeneically transplanted grafts. (A)** ELISA of supernants of cultured allograft-infiltrating cells detected significantly elevated IL-13 levels in allografts (day 60, *P* = 0.0031 and day 100, *P* = 0.0003)compared to syngrafts or FVB control hearts (*P* = 0.0055 and *P* = 0.0042). **(B)** Immunohistochemistry showed significantly higher and over time increasing numbers of IL-13^+^ cells in FVB hearts transplanted into DBA/1 recipients (day 60, *P* = 0.0228; day 100, *P* = 0.0037) relative to syngeneic animals (day 60 versus day 100, *P* = 0.0083). **(C)** Representative immunohistochemical stainings showed more IL-13^+^ cells in allografts ( FVB into DBA/1) compared to controls (DBA/1 into DBA/1; day 100). **(D)** Western blot analysis revealed expression of IL-13Rα_2_ only in allograft-infiltrating cells isolated from allogeneically transplanted hearts (FVB into DBA/1) in contrast to cells isolated from FVB controls or syngrafts (DBA/1 into DBA/1) without IL-13Rα_2_ expression. **(E)** Measurement of TGF-β_1_ by ELISA detected significantly elevated TGF-β_1_ levels in cells isolated from DBA/1 mice grafted with FVB hearts at day 60 (88 ± 5 versus 46 ± 5 pg/mL; *P* = 0.0010) and at day 100 (133 ± 6 versus 42 ± 7 pg/mL; *P* <0.0001) in comparison to syngrafts,and FVB control hearts (40 ± 8 pg/mL; *P* = 0.0048 and *P* = 0.0009, respectively). **(F)** Flow cytometry of graft-infiltrating cells extracted from allografts showed a higher percentage of CD11b^high^Gr1^intermediate^TGF-β_1_^+^ cells (7.3%) than in the syngeneic controls (1.2%; day 100). **(G)** In the flow cytometric analysis (pre-gated for CD45) these CD11b^high^ cells were the only source of TGF-β_1_ production. The histological score is the mean of 3 HPF (20x magnification); at least five mice per group were analyzed. *P <0.05. ELISA, enzyme-linked immunosorbent assay; HPF, high power field; IL-13, interleukin 13; TGF-β1, transforming growth factor beta 1.

To examine if TGF-β_1_ stimulated by IL-13 signaling is elevated in mice receiving allogeneic transplants, IL-13 levels were measured by ELISA in supernatants of cultured allograft-infiltrating cells. Syngeneic DBA/1 heart grafts showed similar IL-13 concentrations at day 60 (108 ± 13 pg/mL) and at day 100 (112 ± 12 pg/mL) (*P* = 0.8415). FVB allografts placed in DBA/1 recipients showed significantly elevated IL-13 levels at day 60 (187 ± 10 pg/mL; *P* = 0.0031) and at day 100 after transplantation (303 ± 23 pg/mL; *P* = 0.0003) in comparison to allogeneic grafts at the same respective time points (Figure 2A). Additionally, immunohistochemical staining for IL-13 in FVB allografts transplanted into DBA/1 mice showed significantly increased numbers of IL-13^+^ cells/HPF at day 60 (16 ± 4 versus 6 ± 2 cells/HPF; *P* = 0.0228) and at day 100 (96 ± 26 versus 7 ± 2 cells/HPF; *P* = 0.0037), relative to the syngeneic controls (day 60 versus day 100; *P* = 0.0083; Figure 2B,C). Western blot analyses of lysates from allograft-infiltrating cells indicated detectable expression of IL-13Rα_2_ only in the allogeneic FVB to DBA/1 mice, both at day 60 and at day 100 after heart transplantation (Figure 2D).

As the next step, the effector cytokine TGF-β_1_ was measured by ELISA after culturing and stimulating cells isolated from the allografts. In DBA/1 mice grafted with FVB hearts, significantly elevated TGF-β_1_ levels were detected at day 60 (88 ± 5 versus 46 ± 5 pg/mL; *P* = 0.0010) and at day 100 (133 ± 6 versus 42 ± 7 pg/mL; *P* < 0.0001) in comparison to the syngeneic controls, and also versus the FVB control heart transplants (40 ± 8 pg/mL; *P* = 0.0048 and *P* = 0.0009, respectively; Figure 2E). In accordance with these results, flow cytometry of graft-infiltrating cells extracted from allogeneic grafts at day 100 showed a higher percentage of CD11b^high^Gr1^intermediate^TGF-β_1_^+^ cells (7.3%) than in the syngeneic controls (1.2%; Figure 2F). Furthermore, flow cytometry demonstrated that these CD11b^high^ cells were likely the only source of TGF-β_1_ production in this transplantation model (Figure 2G).

### FVB allografts transplanted into DBA/1 recipients showed significantly increased levels of collagen deposition

**Figure 3:**
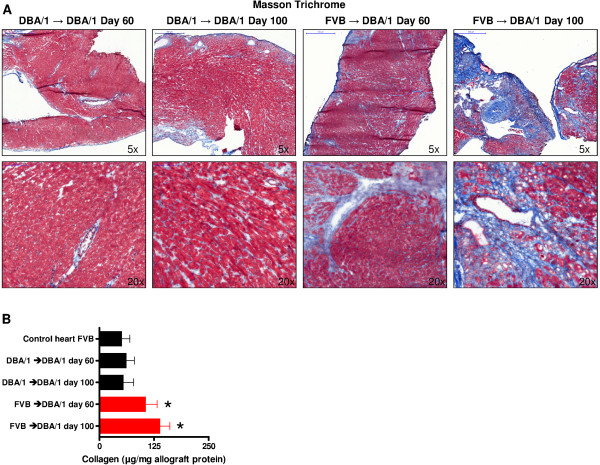
**Increased collagen deposition in allogeneically transplanted grafts. (A)** Representative Masson’s trichrome stainings showed increased levels of collagen (blue color) in allogeneically (FVB into DBA/1) compared to syngeneically (DBA/1 into DBA/1) transplanted hearts explanted at day 60 and day 100 after transplantation (5× and 20× magnification). **(B)** Analysis by Sircol assay detected significantly higher amounts of collagen in FVB hearts placed into DBA/1 recipients at day 60 (*P* = 0.0342) and at day 100 after transplantation (*P* = 0.0022) compared to the DBA/1 to DBA/1 mice and also to FVB control hearts (*P* = 0.0094). At least five mice per group were analyzed. *P < 0.05.

To prove that FVB hearts transplanted in DBA/1 mice develop fibrosis, Masson’s trichrome staining was performed. In these stainings, a strong collagen deposition was found in the allogeneic grafts at day 60, with a further increase in collagen deposition by day 100 after heart transplantation. No such fibrotic collagen deposition was observed in the syngeneic control mice (Figure 3A).

A Sircol assay was conducted to better quantify collagen levels in heart allografts. With this method, the amount of collagen was found to be significantly greater in FVB hearts placed into DBA/1 recipients at day 60 (105 ± 13 versus 61 ± 9 μg/mg allograft protein; *P* = 0.0342) and at day 100 after transplantation (139 ± 11 versus 54 ± 12 μg/mg allograft protein; *P* = 0.0022) compared to the DBA/1-to-DBA/1 mice and also to FVB control hearts (*P* = 0.0094; Figure 3B).

### FVB allografts transplanted into DBA/1 recipients showed upregulation of profibrotic and downregulation of antifibrotic genes

**Figure 4:**
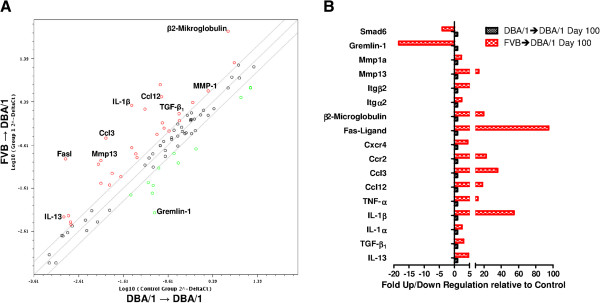
**Activation of a 'fibrotic program’ in allogeneically transplanted grafts. (A,B)** PCR array using RNA extracted from explanted hearts (day 100) of allogeneically (FVB into DBA/1) or syngeneically (DBA/1 into DBA/1) transplanted mice demonstrated an upregulation of pro-fibrotic genes and a downregulation of anti-fibrotic genes in the allogeneic group.

To demonstrate at an mRNA-level that allogeneic grafts have upregulated profibrotic genes, RNA was isolated from the grafted tissues and a PCR array was performed. This PCR array, which profiles the expression of 84 key genes involved in tissue remodeling and fibrosis, revealed an upregulation of IL-13 and TGF-β_1_ (as detected in the previous experiments), but also a strong upregulation of other cytokines relevant for fibrosis, including IL-1α, IL-1β, and TNF-α. Further, chemokines such as Ccl3, Ccl12, Ccr2, and Cxcr4, and genes involved in epithelial-mesenchymal transition or cell adhesion such as Fas ligand, β_2_-microglobulin, integrin-α2, integrin-β6, MMP-1a, and MMP-13, were upregulated; in contrast, Gremlin-1 and Smad6 were downregulated (Figure 4A,B).

**Figure 5:**
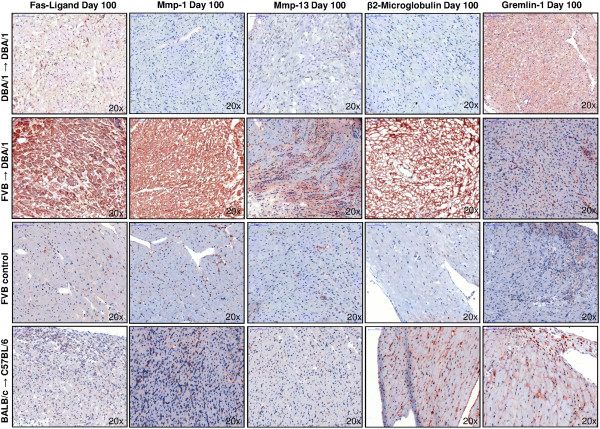
**Immunohistochemical analysis of the 'fibrotic program’ active in allogeneically transplanted grafts.** Representative immunohistochemical stainings show strong positivity for Fas ligand, MMP-1, MMP-13, and β_2_-microglobulin in allogeneically (FVB into DBA/1) and for Gremlin-1 in syngeneically (DBA/1 into DBA/1) transplanted hearts explanted at day 100, in comparison to naive non-transplanted FVB hearts and acutely rejected (BALB/c into C57BL/6) allografts (20× magnification). MMP, matrix metalloproteinase.

Additionally, immunohistochemical labeling was performed for selected targets. According to these PCR array results, FVB allografts placed into DBA/1 recipients showed strong positivity for Fas ligand, MMP-1, MMP-13, and β_2_-microglobulin at day 100, whereas Gremlin-1 staining was stronger in the syngeneic group (Figure 5). In comparison to acutely rejected grafts (BALB/c to C57BL/6), and also to naive non-transplanted FVB hearts, this expression pattern was unique in fibrosis of FVB allografts transplanted into DBA/1 recipients.

### Specific blockage of IL-13Rα_2_ abrogates TGF-β_1_ production and prevents allograft fibrosis

**Figure 6:**
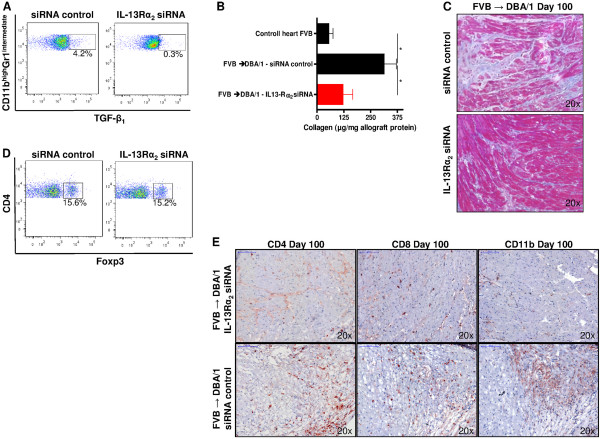
**Blockage of IL-13Rα**_**2 **_**prevents TGF-β**_**1 **_**production and fibrosis in allogeneically transplanted grafts. (A)** Flow cytometric analysis of graft-infiltrating cells of allogeneically (FVB into DBA/1) transplanted hearts (day 100) detected a strongly diminished percentage of CD11b^high^Gr1^intermediate^TGF-β_1_^+^ cells (0.3%) in the siRNA-treated group compared to the control animals (4.2%). **(B)** Quantification of collagen by Sircol assay showed significantly less collagen levels in mice injected with specific IL-13Rα_2_ siRNA (*P* = 0.0018) than mice treated with control siRNA. Levels were not significantly different to FVB control hearts (*P* = 0.1309). **(C)** Representative Masson’s trichrome stainings showed reduced levels of collagen in allogeneically (FVB into DBA/1) transplanted hearts treated with siRNA compared to mice treated with control peptide (day 100 after transplantation; 20x magnification). **(D)** In flow cytometric analysis the frequency of CD4^+^Foxp3^+^ Tregs isolated from allogeneically transplanted grafts (day 100) was similar in siRNA- and control siRNA-injected animals (15.6 versus 15.2%). **(E)** Representative immunohistochemical stainings (day 100) showed numbers of CD4^+^, CD8^+^, and CD11b^+^ cells in FVB allografts transplanted into DBA/1 mice and treated with siRNA that were lower than mice treated with control siRNA and comparable to syngeneic control grafts (Figure 1 A,B,C). At least five mice per group were analyzed. *P <0.05. Treg, regulatory T cell.

To investigate if allograft fibrosis depends on TGF-β_1_ production stimulated by IL-13 secretion, IL-13 signaling in DBA/1 recipients grafted with FVB hearts was inhibited by intraperitoneal treatment with specific IL-13Rα_2_ siRNA or control siRNA. Flow cytometric analysis of graft-infiltrating cells from hearts harvested 100 days after transplantation showed a low percentage of CD11b^high^Gr1^intermediate^TGF-β_1_^+^ cells (0.3%) in the siRNA-treated group compared to controls (4.2%; Figure 6A). Furthermore, quantification of collagen by the Sircol assay showed significantly less collagen levels in mice treated with specific IL-13Rα_2_ siRNA compared to mice treated with control siRNA (122 ± 23 versus 314 ± 28 μg/mg allograft protein; *P* = 0.0018) (Figure 6B). This was in accordance with Masson’s trichrome staining, in which only small areas of collagen deposition were observed in siRNA-treated hearts, whereas extensive amounts of collagen were detected in control allografts (Figure 6C). To test whether the differences in TGF-β_1_ production caused imbalances in CD4^+^Foxp3^+^ regulatory T cells (Tregs), a flow cytometric analysis of cells from allografts was performed 100 days after transplantation. However, CD4^+^Foxp3^+^ Tregs were found to be present at equal levels in siRNA- and control siRNA-injected animals (15.6% versus 15.2%, respectively; Figure 6D). Immunohistochemical staining of allografts after therapy with specific IL-13Rα_2_ siRNA showed levels of CD4^+^, CD8^+^, and CD11b^+^ cells that were much lower after treatment with control siRNA, but similar to syngeneic transplanted animals (Figure 6E versus Figure 1A,B,C).

In this study we demonstrate for the first time that allograft fibrosis is caused by IL-13 signaling through the receptor IL-13Rα_2_, which consequently leads to elevated TGF-β_1_ levels resulting in increased collagen deposition in heart allografts. Additionally, we show that inhibition of this pathway by siRNA specific for IL-13Rα_2_ prevents allograft fibrosis.

The findings presented here that link IL-13 signaling via IL-13Rα_2_ to allograft fibrosis are based on our previous studies showing that such signaling is essential in the development of inflammation-associated fibrosis [9,10,17]. These studies showed that IL-13 induces TGF-β_1_ via a two-stage process involving: 1) induction of IL-13Rα_2_ expression by IL-13 (or IL-4) signaling via IL-13Rα_1_, combined with TNF-α signaling through its receptor; and 2) IL-13 signaling via IL-13Rα_2_ to induce an AP-1 variant containing c-Jun and Fra-2 that activates the TGF-β1 promoter [9]. The importance of this pathway for development of fibrosis has been shown extensively by our group in bleomycin-induced lung fibrosis and chronic TNBS-induced colitis [10,17]. Thus, these previous studies provided the basis to investigate the importance of IL-13/TGF-β_1_ signaling in the setting of allograft fibrosis.

The study presented here shows increasing levels of IL-13 and IL-13^+^ cells within allografts of transplanted mice, in contrast to control mice receiving syngeneic grafts. Multiple studies have demonstrated that IL-13 is essential for the development of dermal, gastrointestinal, and pulmonary fibrosis, as well as fibro-obliterative lesions found in the bronchiolitis obliterans (BO) syndrome [9,10,17-20]. Consistent with these studies, IL-13Rα_2_ was detected only in the FVB allografts transplanted into DBA/1 recipients in our experiments. This receptor has been shown to link IL-13 signaling with further fibrotic downstream effects [9,21]. In follow-up, we detected elevated levels of TGF-β_1_ in the mice receiving allogeneic grafts exclusively. Results from previous studies have indicated that TGF-β_1_ is the key cytokine for development of allograft fibrosis in murine models and in humans, and that depletion of TGF-β_1_ can prevent allograft fibrosis [7,12,22]. Other cytokines such as IL-6 and IL-17 can modulate the TGF-β_1_-mediated fibrotic reactions [7,8]. Additionally, a study by Faust *et al*. concluded that T cell TGF-β signaling was required for the development of allograft fibrosis [8]. In parallel to the elevated levels of TGF-β_1_, we found increased allograft-infiltration with CD11b^high^Gr1^intermediate^TGF-β_1_^+^ cells in the DBA/1 mice transplanted with FVB allografts; it has been shown by our group and others that CD11b^high^Gr1^intermediate^ cells are the main source for TGF-β_1_ production [23-25]. In the allogeneic situation of the mouse model we used, activation of the profibrotic IL-13/TGF-β_1_ interaction led to allograft fibrosis that was continuously increasing over time after transplantation.

Another important finding from this study is that allograft fibrosis can be prevented by blockage of the IL-13/TGF-β_1_ interaction through specific IL-13Rα_2_ siRNA. After treatment with IL-13Rα_2_ siRNA, an almost complete reduction of TGF-β_1_ production by CD11b^high^Gr1^intermediate^ cells (the main producers of TGF-β_1_ in this model) was observed [24,25]. The reduction of TGF-β_1_-producing cells and reduced TGF-β_1_ levels consequently led to diminished collagen deposition in heart allografts and therefore reduced allograft fibrosis. Tregs were also considered as contributors to the TGF-β_1_ effect. While CD4^+^Foxp3^+^ Tregs can produce TGF-β_1_ to mediate their tolerogenic functions and expand induced regulatory T cells (iTregs), there was no difference in their numbers in control versus IL-13Rα_2_ siRNA-injected mice [26-29]. Notably, after therapy with IL-13Rα_2_ siRNA, CD4^+^ and CD8^+^ cells were found at levels that were similar to mice receiving syngeneic grafts, and were much lower than in allotransplanted mice not given IL-13Rα_2_ siRNA treatment.

For our investigations, we used a heterotopic murine heart transplantation model in which FVB hearts were placed in DBA/1 recipients. This chronic rejection model with minor multiple non-MHC mismatches has been used previously to study graft coronary artery disease [14]. We show that the FVB to DBA/1 model can also be used to examine allograft fibrosis. Over time, transplanted allografts are infiltrated by increasing numbers of CD4^+^, CD8^+^, and CD11b^+^ cells, a fact that was also observed by Tanaka *et al*. in the original description of this transplantation model [14]. We further demonstrated by PCR array that a 'fibrotic program’ is active in this FVB to DBA/1 model. Profibrotic factors such as IL-1α and IL-1β that play a role in liver fibrosis development, and TNF-α which is an essential cofactor of IL-13 to induce the expression of IL-13Rα_2_, were upregulated. Further, Ccl-12 and Cxcr-4 that were both shown to be involved in pulmonary fibrosis, Ccl-3 which has been described to be important in systemic sclerosis, and Ccr-2 that is associated with allograft fibrosis, were overexpressed [9,30-33]. Additionally, the PCR array showed upregulation of other influential molecules such as Fas/Fas ligand, which is important in the development of fibrotic lesions associated with adult respiratory distress syndrome (ARDS). MMP-1 and MMP-13 (involved in remodeling processes occurring during fibrosis) and β2-microglobuin were also overexpressed in the PCR array and positive in the immunohistochemistry of FVB allografts transplanted into DBA/1 recipients [34-36]. In contrast, genes like Gremlin-1 that may contribute to reversibility of lung fibrosis in rats, and Smad6 which in complex with Smurf-1 effectively attenuated TGF-β_1_ signaling, were downregulated [37,38]. Altogether, these findings support the fact that this FVB to DBA/1 transplantation model is suitable not only to study graft coronary artery disease, but also to examine organ allograft fibrosis.

## Conclusions

In conclusion, this study shows that IL-13 signaling via IL-13Rα_2_ induces TGF-β_1_ and causes allograft fibrosis in a chronic transplant rejection model that is now also established as a model to study allograft fibrosis. Further, we demonstrate in this study that blockage of this IL-13/TGF-β_1_ interaction by IL-13Rα_2_ siRNA prevents heart allograft fibrosis. Together, our results indicate that IL-13Rα_2_ may be exploitable as a future target to reduce allograft fibrosis in organ transplantation.

## Abbreviations

ACK: Ammonium-chloride-potassium; ARDS: Adult respiratory distress syndrome; BO: Bronchiolitis obliterans; BSA: Bovine serum albumin; DAB: 3,3'-diaminobenzidine; DNase: Deoxyribonuclease I; ELISA: Enzyme-linked immunosorbent assay; FCS: Fetal calf serum; HBSS: Hanks’ balanced salt solution; HPF: High power field; HVJ-E: Hemagglutinating virus of Japan envelope; IgG: Immunoglobulin G; IL-13: Interleukin 13; iTreg: Induced regulatory T cell; MHC: Major histocompatibility complex; MMP: Matrix metalloproteinase; PCR: Polymerase chain reaction; RPMI: Roswell park memorial institute; SEM: Standard error of the mean; siRNA: Small interfering RNA; TGF-β1: Transforming growth factor beta 1; TNBS: 2,4,6-trinitrobenzene sulfonic acid; TNF: Tumor necrosis factor; Treg: Regulatory T cell

## Competing interests

The authors declare that they have no competing interests.

## Authors’ contributions

SMB designed the study concept, collected and analyzed data, and wrote the manuscript. GS, RK, SB, and MM collected and analyzed data. HJS and EKG analyzed data and reviewed the manuscript. SFF designed the study concept, collected and analyzed data, and reviewed the manuscript. All authors read and approved the final manuscript.
